# Decentralising atrial fibrillation screening to overcome socio-demographic inequalities in uptake in STROKESTOP II

**DOI:** 10.1177/0969141320908316

**Published:** 2020-03-30

**Authors:** Katrin Kemp Gudmundsdottir, Anders Holmen, Tove Fredriksson, Emma Svennberg, Faris Al-Khalili, Johan Engdahl, Ulf Strömberg

**Affiliations:** 1 Department of Clinical Sciences, Danderyd University Hospital, Karolinska Institutet, Stockholm, Sweden; 2 Department of Research and Development, Region Halland, Halmstad, Sweden; 3 Health Metrics Unit, Institute of Medicine, Sahlgrenska Academy at University of Gothenburg, Gothenburg, Sweden

**Keywords:** Screening uptake, atrial fibrillation, socioeconomic factors

## Abstract

**Objective:**

In the first STROKESTOP atrial fibrillation screening study, participation was influenced by socio-demographic and geographic factors. To improve uptake in the second study, two screening sites were added, closer to low-income neighbourhoods which had very low participation in the first study. This paper aims to analyse the geographic and socio-demographic disparities in uptake in the second trial and compare the results with the first trial.

**Methods:**

Inhabitants of the Stockholm region born in 1940 and 1941 were randomised 1:1 to be invited to screening or serve as controls. Medical history, blood samples and single-lead-ECG were collected. Invitee’s residential parish was used for geo-mapping analysis of the geographical disparities in participation, using hierarchical Bayes methods. Individual data for participants and non-participants were obtained for the socioeconomic variables: educational level, disposable income, immigrant and marital status.

**Results:**

Higher participation was observed in those with higher education, high income, among non-immigrants and married individuals. Participation between the first and second studies improved significantly, where additional screening sites were introduced. These improvements were generally significant, in each population group according to socio-demographic characteristics.

**Conclusion:**

Decentralisation of screening sites in an atrial fibrillation screening program yielded a significantly positive impact on screening uptake. Adding local screening sites in areas with low uptake had beneficial impact on participation across a wide spectrum of socio-demographic groups. Decentralised screening substantially increased the screening uptake in deprived areas.

## Introduction

Atrial fibrillation (AF) is the most common sustained cardiac arrhythmia and a major cause of cardiovascular mortality and morbidity.^1^ In Sweden, it affects at least 2.9% of the population aged >20. Prevalence increases with age, reaching 9.7% at age 70–79.^2^ The global burden of AF is likely to increase, as the prevalence is expected to double within the next 30 years due to expected demographic shifts.^3^ AF is a known risk factor for heart failure, dementia and death,^4^,^5^ as well as ischaemic stroke.^6^ In high-risk patients, stroke risk can be reduced by at least two-thirds with oral anticoagulant therapy.^7^ Stroke can be the first clinical manifestation of AF, as AF can be both asymptomatic and intermittent.^8^ Screening for AF has been proposed in the European Society of Cardiology guidelines, and in the AF-SCREEN International Collaboration white paper from 2017, although large, randomised outcome studies are still needed to strengthen this case.^9^

An important factor for screening success is uptake. Previous studies have shown that the likelihood of attending a screening program is correlated to socioeconomic status.^10,11^ In a sub-study to the STROKESTOP study (SS1), in which socio-demographic differences of the participants were studied, participation was significantly influenced by socio-demographic factors.^12^ Lower education level, lower income level and immigrant status were all associated with lower uptake. Gender did not influence participation. Geographical inequalities in the screening participation beyond socio-demographic characteristics were also observed. In investigating new strategies to address inequalities in screening uptake, preventive efforts in socioeconomically disadvantaged neighbourhoods may be worthwhile.^13^

The findings from SS1, a study of AF-screening among Swedish participants aged 75,^14^ were considered when designing the STROKESTOP II study (SS2) protocol.^15^ One major intervention was decentralised screening. Two screening sites were added in SS2, located closer to low-income neighbourhoods with a very low participation in SS1.

This study aimed to analyse geographic and socio-demographic disparities in the uptake of the SS2 study and compare the results with those from SS1.

## Methods

**Figure 1.**
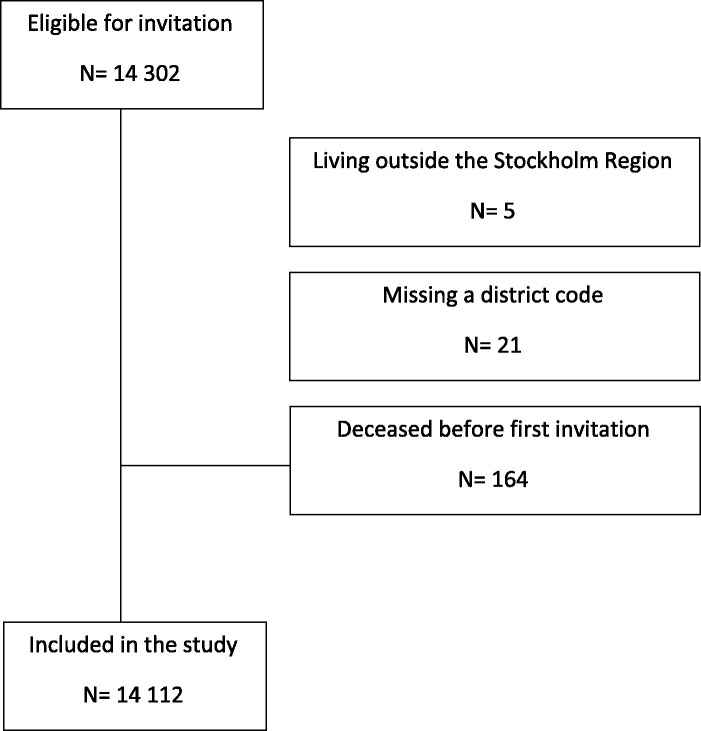
Study inclusion flow chart.

The SS2 study design has previously been published.^15^ Briefly, half of all inhabitants in the Stockholm region born 1940 and 1941 were identified using their personal identification number by Statistics Sweden. A stratified, gender- and age-based 1:1 randomisation provided an intervention group to be invited to participate in an AF screening study, while the other half served as a control group. No information or intervention was provided to the control group. The intervention group was invited to screening via mail with a maximum of two reminders for non-responders. The only inclusion criteria were birth year and residence in the Stockholm region for those in the intervention group; there were no exclusion criteria. The intention-to-screen arm comprised 14,112 persons, and data were collected between April 2016 and February 2018. Participants received oral and written information, and signed informed consent documents. Participants reported their medical history, and those without previous AF had NT-proBNP analysed from venous blood samples and recorded a 30 s, handheld single-lead-ECG using the Zenicor II device (Zenicor Medical Systems, Stockholm, Sweden). Depending on the NT-proBNP results, participants were stratified to either prolonged ECG screening, consisting of two-week intermittent ambulatory handheld ECG recordings using the Zenicor II device, or to no further ECG screening. The results from the AF screening have been published.^16^ This study addressed participation among those who were invited; data from the control group were not relevant. The study flow chart is shown in Figure 1.

To improve screening uptake, a website (www.strokestop2.se) was launched providing general information on AF, the study procedure and the study team. Patient information was translated to the nine most common languages in Sweden. Three different screening sites were used to shorten the travel distance for participants. The Sabbatsberg Hospital site was the same as used in SS1. The two new sites in SS2 were Södertälje Hospital and Karolinska University Hospital.

The SS2 database comprises information on each invited person’s residential parish (99 parishes in Stockholm). Statistics Sweden provided both participants and non-participants with anonymised individual data for each of the following socioeconomic variables: educational level classified based on the number of school years completed (≤9 years, i.e. primary school; ≥10 years, i.e. secondary school/pre university/university), disposable income (<15,000 Euro/year, referred to as “low”; 15,000–30,000 Euro/year, “medium”; >30,000 Euro/year, “high”), immigrant (born in Sweden, born abroad) and marital status (unmarried, married, divorced, widow/widower). Invited persons who could not be classified based on the information in the national registers were grouped into an “other/unknown” category of the variable at issue. The same socio-demographic variables were considered in SS1, but we did not obtain individual-level data on the socio-demographic characteristics. The data used in SS1 were aggregated at parish level.

*p*-Values for the null hypothesis of equal participation in men and women and for each socioeconomic variable were obtained by chi-square test. All tests were two-sided, and a value of *p* < 0.05 was considered significant.

Geo-maps of Stockholm county displaying spatially smoothed participation ratios (PRs) were estimated by hierarchical Bayes methods. A parish-specific PR is based on the observed-to-expected numbers of participants, where the expected number was obtained from the sex-specific participation rates for the total study population of the county. Spatially smoothed PRs were obtained by running the hierarchical Bayesian mapping model (the Besag-York-Mollié model) implemented in the Rapid Inquiry Facility program.^17^ This procedure allows parish-specific participation rates to be smoothed towards global and local mean participation rates across the county, yielding “shrinkage” of the conventional observed to expected ratios—in line with principles for multi-level modeling.^18^ The corresponding statistical certainty geo-maps were obtained by calculating the posterior probabilities of a PR > 1 given the data, denoted Pr (PR > 1|data), using the Bayesian approach.

A parish with data yielding strong statistical evidence of elevated participation, more precisely Pr (PR >1|data)> 0.90, was coloured green in the certainty geo-map. By contrast, a parish with lowered participation rate, Pr (PR < 1|data) >0.90, was coloured red. The remaining parishes were coloured yellow. The choice of 0.90 for identifying an area with elevated/lowered participation rate has been shown to provide a cut-off with reasonable sensitivity and high specificity.^19^

Binary logistic regression was used for the univariate and multivariable analyses of socio-demographic factors for the outcome reflecting participation or non-participation.

The statistical computations were performed by using IBM SPSS Statistics for Windows, Version 24.0. Armonk, NY: IBM Corp. For the spatial analyses, the Rapid Inquiry Facility free software (RIF 3.12).^17^

The study complies with the Declaration of Helsinki, and the protocol was approved by the regional ethics committee (DNR 2015/2079–31/1). Informed consent was obtained from all participants in the screening program. ClinicalTrials.gov identifier: NCT02743416.

## Results

**Figure 2.**
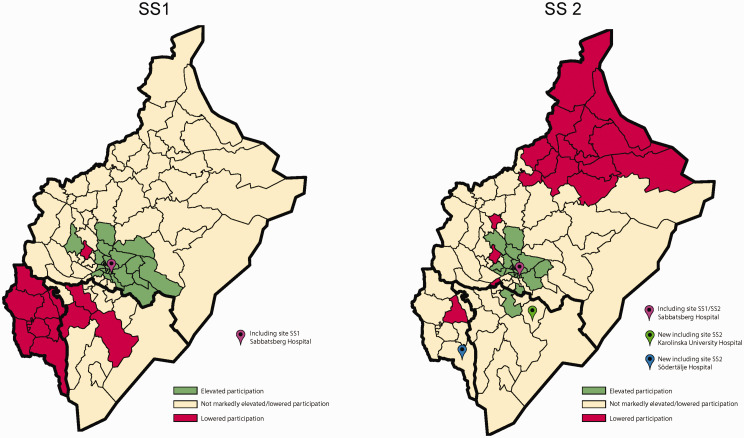
Statistical certainty geo-maps of participation in the SS1 study and the SS2 study in Stockholm county.

Overall participation was 48.6%, a significant increase (*p* = 0.006) from the 46.9% uptake in SS1 in the Stockholm region. Figure 2 shows the statistical certainty geo-maps of PRs in the SS1 study and the SS2 study in Stockholm county displaying, for each of the 99 residential parishes, the spatially smoothed PRs which were evidently above or below 1. The addition of the two new southern Stockholm sites in SS2 increased the participation rates in those areas. In comparison, the northern region is depicted as red in SS2. This was due to increased uptake in the southern part, not because of lower actual uptake rates between the STROKESTOP trials.

Compared with the same catchment areas and corresponding PRs from SS1, SS2 participation rates were significantly higher in the areas where a screening site had been added. In the area surrounding the Karolinska University Hospital site, uptake increased from 41% to 47% (*p* < 0.001), a 15% relative increase. In the vicinity of the Södertälje Hospital site, uptake increased from 28% to 43% (*p* < 0.001), a 54% relative increase. Uptake was not significantly changed (50% in SS1 vs. 49% in SS2, *p* = 0.17) in the catchment area of the Sabbatsberg Hospital site (the one site used in both studies, but with a smaller catchment area in SS2). In all three catchment areas, higher participation was observed among women, those with higher education, those with high income, among non-immigrants and among married individuals (see Table 1). Uptake was improved most markedly in the area around the Södertälje Hospital site, where a 1.5-fold increase was observed also in the socioeconomically weaker population groups (from 21% to 32% participants in the low education group, 22% to 32% participants in the low-income group and 31% to 47% participants in the immigrant group). The increases in uptake were pronounced (at least 10 percentage points) in socially deprived population groups with very low participation in SS1.

**Table 1.** Participation rates in SS2 according to catchment area and socio-demographic characteristics, compared with corresponding rates in SS1.

	Karolinska University Hospital				Sabbatsberg Hospital				Södertälje Hospital			
	Invited (n)	Participants (n)	%	*p* ^a^	Invited (n)	Participants (n)	%	*p* ^a^	Invited (n)	Participants (n)	%	*p* ^a^
Total												
SS1	1714	705	41		9517	4786	50		618	174	28	
SS2	2086	983	47	<0.001	11,240	5544	49	0.17	786	334	43	<0.001
By gender												
Male SS1	802	342	43		4311	2144	50		285	84	29	
Male SS2	976	443	45	0.26	5306	2565	48	0.18	346	145	42	0.002
Female SS1	912	363	40		5260	2642	50		333	90	27	
Female SS2	1110	540	49	<0.001	5934	2979	50	0.99	440	189	43	<0.001
By educational level^b^												
Primary school SS1	585	198	34		2573	1009	39		267	56	21	
Primary school SS2	615	235	38	0.13	2475	912	37	0.09	262	85	32	0.004
Secondary school/higher SS1	1077	497	46		6759	3727	55		303	108	36	
Secondary school/higher SS2	1414	739	52	0.003	8513	4584	54	0.11	470	243	52	<0.001
By disposable income^c^												
Low SS1	964	334	35		4258	1681	39		389	86	22	
Low SS2	854	328	38	0.11	3354	1181	35	<0.001	369	119	32	0.002
Medium SS1	647	315	49		4066	2328	57		189	70	37	
Medium SS2	989	509	51	0.29	5513	2900	53	<0.001	347	176	51	0.003
High SS1	100	54	54		1232	775	63		40	18	45	
High SS2	243	146	60	0.36	2373	1463	62	0.48	70	39	56	0.38
By immigrant background												
Born in Sweden SS1	1199	527	44		7565	4021	53		432	136	31	
Born in Sweden SS2	1551	795	51	<0.001	8952	4690	52	0.34	517	243	47	<0.001
Born outside of Sweden SS1	515	178	35		2006	765	38		186	38	20	
Born outside of Sweden SS2	535	188	35	0.90	2288	854	37	0.61	269	91	34	0.003
By marital status^d^												
Married SS1	948	435	46		5077	2786	55		311	106	34	
Married SS2	1194	617	52	0.009	5981	3209	54	0.21	427	200	47	<0.001
Divorced SS1	354	138	39		2011	906	45		131	32	24	
Divorced SS2	420	175	42	0.49	2626	1172	45	0.80	169	73	43	0.001
Widow/widower SS1	311	107	34		1657	762	46		134	28	21	
Widow/widower SS2	320	132	41	0.09	1499	684	46	0.87	134	46	34	0.02
Unmarried SS1	101	25	25		819	325	40		42	8	19	
Unmarried SS2	152	59	39	0.03	1134	479	42	0.28	56	15	27	0.51

SS2: STROKESTOP 2 Study; SS1: STROKESTOP 1 Study.

^a^*p*-value based on the chi-squared test, comparing the participation rates between SS2 and SS1 for a given population group within the specified catchment area.

^b^Data on educational level were missing for 339 invited persons in SS1 and 363 invited persons in SS2.

^c^Data on disposable income were missing for 18 invited persons in SS1.

^d^Data on marital status were missing for seven invited persons in SS1.

A univariate logistic regression analysis showed that the odds of participation in SS2 were highest among women, those with higher education, those with high incomes, non-immigrants, married people and those living in the catchment area belonging to the Sabbatsberg Hospital site. The difference in odds ratio in all categories compared with reference was significant. In the multivariable analysis, the odds of attendance were consistent with those in the univariate analysis, except in the catchment area where the difference became insignificant (see Table 2).

**Table 2.** Results from logistic regression analyses based on data from SS2.

Variable	Univariate analyses					Multivariable analysis	
	Invited (n)	Participants (n)	%	OR (95% CI)	*p* ^a^	OR (95% CI)	*p* ^a^
Gender					0.02		<0.001
Men	6628	3153	47.6	1.00 (ref)		1.00 (ref))	
Women	7484	3708	49.5	1.08 (1.01, 1.16)		1.43 (1.32, 1.54)	
Educational level					<0.001		<0.001
Primary school	3352	1232	36.8	1.00 (ref)		1.00 (ref)	
Secondary school/higher	10,397	5566	53.5	1.98 (1.83, 2.14)		1.65 (1.52, 1.79)	
Unknown	363	63	17.4	0.36 (0.27, 0.48)		0.50 (0.37, 0.66)	
Disposable income					<0.001		<0.001
Low	4577	1628	35.6	1.00 (ref)		1.00 (ref)	
Medium	6849	3585	52.3	1.99 (1.84, 2.15)		1.91 (1.75, 2.07)	
High	2686	1648	61.4	2.88 (2.61, 3.17)		2.58 (2.31, 2.88)	
Immigrant background					<0.001		<0.001
Born outside Sweden	3092	1133	36.6	1.00 (ref)		1.00 (ref)	
Born in Sweden	11,020	5728	52.0	1.87 (1.72, 2.03)		1.36 (1.24, 1.48)	
Marital status					<0.001		<0.001
Unmarried	1342	553	41.2	1.00 (ref)		1.00 (ref)	
Widow/widower	1953	862	44.1	1.13 (0.98, 1.30)		0.97 (0.84, 1.13)	
Divorced	3215	1420	44.2	1.13 (0.99, 1.28)		1.06 (0.93, 1.21)	
Married	7602	4026	53.0	1.61 (1.43, 1.81)		1.56 (1.38, 1.76)	
Catchment area					<0.001		0.59
Södertälje Hospital	786	334	42.5	1.00 (ref)		1.00 (ref)	
Karolinska University Hospital	2086	983	47.1	1.21 (1.02, 1.42)		1.07 (0.90, 1.27)	
Sabbatsberg Hospital	11,240	5544	49.3	1.32 (1.14, 1.52)		1.02 (0.87, 1.19)	

SS2: STROKESTOP 2 Study.

^a^*p*-value for variable.

## Discussion

This study illustrates that geographic distance to the screening site is of high importance, and that increasing the number of sites has the potential to significantly increase uptake in screening studies. Socio-demographic factors have a significant impact on AF screening uptake. The increased uptake due to decentralised screening was particularly notable in attendees with socio-demographic factors which were previously associated with higher odds of attending. Notably, we also observed markedly increased participation in population groups with low socioeconomic status. The outcome of efforts to increase participation in socioeconomically weak groups was most pronounced in the catchment area of the Södertälje Hospital site, where the participation was very low in SS1. These results indicate that decentralised screening can substantially increase uptake in deprived areas. This site is further away from the site used in SS1 than the other new site, Karolinska Hospital, which could be one reason for the larger increase in uptake observed around the Södertälje site.

These results reinforce the importance of geographic proximity in screening. We found that screening uptake in those normally not attending screening (i.e. with the lowest educational level and income) might be affected by proximity of screening sites. Lower participation in this screening study goes hand in hand with increasing socio-demographic deprivation, which confirms the results from a previous study in which men aged 65 were invited to abdominal aortic aneurysm screening and those with lower socioeconomic status showed lower compliance.^11^ A recent systematic review found inconsistent evidence for lower socioeconomic status and AF diagnosis, but those with lower socioeconomic status showed poorer outcomes when AF was present.^20^ A study of four different strategies to reduce the socioeconomic gradient of uptake in the English NHS Bowel Cancer Screening Program found only an enhanced reminder letter to have an effect on the socioeconomic gradient.^21^ These results stress the need for targeted actions to increase uptake in those with lower socioeconomic status, although a systematic review found that barriers and facilitators to participation in health checks for cardiometabolic disease were heterogenous, which makes it difficult to develop a “one size fits all” approach for increased uptake.^22^

Travel distance to the screening site probably influences participation, as the increase in participation was markedly higher in the uptake areas of the two new sites compared with the area around the one site used in both STROKESTOP trials. The area with the lowest participation rates in SS2 was the one farthest from any screening site (Figure 2). In a Danish study on screening for vascular disease, specially trained nurses operated mobile clinics in hospitals, general practitioners’ offices and even in a town hall, resulting in an uptake of 74.7%.^23^

All invitees in SS2 were aged 75–76, and there were no exclusion criteria. This could possibly mean that some invitees were not able to attend (i.e. in assisted living facilities, severe disabilities or dementia diagnosis), and thus the sampling frame should possibly be smaller than it is in this study.

As this is a study on AF screening, and not an established screening program, some non-responders may have been interested in participating in an established routine screening program, but not in a clinical trial. The European Society of Cardiology 2016 Guidelines for the management of AF gives a class Ib recommendation for opportunistic screening, and a IIb recommendation for systematic screening in individuals aged >75 or those at high stroke risk. This is based on studies showing increased detection of previously unknown AF in individuals with screening and the fact that the connection between AF and stroke in high-risk individuals is well established.^1^ No study has shown that screening for AF reduces stroke incidence, although several are ongoing, including the STROKESTOP trials. Due to the lack of evidence, the US Preventive Services Task force recommendation statement on screening for AF from 2018 does not recommend population screening for AF and concludes that the current evidence is insufficient to assess the balance of benefits and harms of screening for AF.^24^ Similarly, the 2019 UK National Screening Committee concluded that screening for AF is not recommended, based on a lack of trials that compare formal screening with routine clinical diagnosis, and that evaluate clinical health outcomes.^25^

Our study has some limitations. The comparison of the two STROKESTOP trials is a comparison of two different study populations, as the studies and individual data collection are performed a few years apart. Therefore, the participants are not the same, and the demographics of the areas may have changed in these years, potentially introducing misclassification bias. Noticeably, there seems to have been a systematic change in the disposable income category, with a lower proportion of individuals in the lowest income category in SS2 compared with SS1, a shift that makes the comparison of the studies potentially biased. Such a bias would mean that we would detect a difference that reflects a change in demographics, rather than in participation. In addition, the effects of changes (website, more languages in the patient information, more sites) made to increase the screening uptake cannot be separated. This study cannot answer which changes had the strongest impact, and this may decrease the study’s external validity.

## Conclusion

Decentralisation of screening sites in an AF screening program yielded a significantly positive impact on screening uptake. The addition of local screening sites in areas with low uptake had a beneficial impact on participation across a wide spectrum of socio-demographic groups. Importantly, decentralised screening increased substantially the screening uptake in deprived areas.

## References

[1] Kirchhof P Benussi S Kotecha D, et al ESC Guidelines for the management of atrial fibrillation developed in collaboration with EACTS: the Task Force for the management of atrial fibrillation of the European Society of Cardiology (ESC) developed with the special contribution of the European Heart Rhythm Association (EHRA) of the ESC Endorsed by the European Stroke Organisation (ESO). Eur J Cardio-Thoracic Surg 2016; 50: e1–e88. 10.1093/ejcts/ezw313 27663299

[2] Friberg L Bergfeldt L. Atrial fibrillation prevalence revisited. J Intern Med 2013; 274: 461–468. 23879838 10.1111/joim.12114

[3] Krijthe BP Kunst A Benjamin EJ, et al Projections on the number of individuals with atrial fibrillation in the European Union, from 2000 to 2060. Eur Heart J 2013; 34: 2746–2751. 23900699 10.1093/eurheartj/eht280 PMC3858024

[4] Healey JS Oldgren J Ezekowitz M, et al Occurrence of death and stroke in patients in 47 countries 1 year after presenting with atrial fibrillation: a cohort study. Lancet 2016; 388: 1161–1169. 27515684 10.1016/S0140-6736(16)30968-0

[5] Singh-Manoux A Fayosse A Sabia S, et al Atrial fibrillation as a risk factor for cognitive decline and dementia. Eur Heart J 2017; 38: 2612–2618. 28460139 10.1093/eurheartj/ehx208 PMC5837240

[6] Wolf PA Abbott RD Kannel WB. Atrial fibrillation as an independent risk factor for stroke: the Framingham Study. Stroke 1991; 22: 983–988. 1866765 10.1161/01.str.22.8.983

[7] Hart RG Pearce LA Aguilar MI. Meta-analysis: antithrombotic therapy to prevent stroke in patients who have nonvalvular atrial fibrillation. Ann Intern Med 2007; 146: 857–867. 17577005 10.7326/0003-4819-146-12-200706190-00007

[8] Lubitz SA Yin X McManus DD, et al Stroke as the initial manifestation of atrial fibrillation: the Framingham Heart Study. Stroke 2017; 48: 490–492. 28082669 10.1161/STROKEAHA.116.015071 PMC5262530

[9] Freedman B Camm J Calkins H, et al Screening for atrial fibrillation: a report of the AF-SCREEN International Collaboration. Circulation 2017; 135: 1851–1867. 28483832 10.1161/CIRCULATIONAHA.116.026693

[10] Maheswaran R Pearson T Jordan H, et al Socioeconomic deprivation, travel distance, location of service, and uptake of breast cancer screening in North Derbyshire, UK. J Epidemiol Community Health 2006; 60: 208–212. 16476749 10.1136/jech.200X.038398 PMC2465550

[11] Zarrouk M Holst J Malina M, et al The importance of socioeconomic factors for compliance and outcome at screening for abdominal aortic aneurysm in 65-year-old men. J Vasc Surg 2013; 58: 50–55. 23541548 10.1016/j.jvs.2012.12.080

[12] Engdahl J Holmen A Svennberg E, et al Geographic and socio-demographic differences in uptake of population-based screening for atrial fibrillation: the STROKESTOP I study. Int J Cardiol 2016; 222: 430–435. 27505329 10.1016/j.ijcard.2016.07.198

[13] Stromberg U Holmen A Engdahl J. Comprehensive analyses of geographical inequalities in cardiovascular disease incidence and survival: a possible aid to rationalize prevention strategies. Eur J Prev Cardiolog 2019; 26: 1824–1827. 10.1177/2047487319856940 31180760

[14] Svennberg E Engdahl J Al-Khalili F, et al Mass screening for untreated atrial fibrillation: the STROKESTOP study. Circulation 2015; 131: 2176–2184. 25910800 10.1161/CIRCULATIONAHA.114.014343

[15] Engdahl J Svennberg E Friberg L, et al Stepwise mass screening for atrial fibrillation using N-terminal pro b-type natriuretic peptide: the STROKESTOP II study design. Europace 2016; 19: 297–302. 10.1093/europace/euw319 28011798

[16] Kemp Gudmundsdottir K Fredriksson T Svennberg E, et al Stepwise mass screening for atrial fibrillation using N-terminal B-type natriuretic peptide: the STROKESTOP II study. Europace 2019; 22: 24–32. 10.1093/europace/euz255 PMC6945054 31790147

[17] Beale L Abellan JJ Hodgson S, et al Methodologic issues and approaches to spatial epidemiology. Environ Health Perspect 2008; 116: 1105–1110. 18709139 10.1289/ehp.10816 PMC2516558

[18] Greenland S. Principles of multilevel modelling. Int J Epidemiol 2000; 29: 158–167. 10750618 10.1093/ije/29.1.158

[19] Richardson S Thomson A Best N, et al Interpreting posterior relative risk estimates in disease-mapping studies. Environ Health Perspect 2004; 112: 1016–1025. 15198922 10.1289/ehp.6740 PMC1247195

[20] Lunde ED Nielsen PB Riahi S, et al Associations between socioeconomic status, atrial fibrillation, and outcomes: a systematic review. Expert Rev Cardiovasc Ther 2018; 16: 857–873. 30293472 10.1080/14779072.2018.1533118

[21] Wardle J von Wagner C Kralj-Hans I, et al Effects of evidence-based strategies to reduce the socioeconomic gradient of uptake in the English NHS Bowel Cancer Screening Programme (ASCEND): four cluster-randomised controlled trials. Lancet 2016; 387: 751–759. 26680217 10.1016/S0140-6736(15)01154-X PMC4761689

[22] de Waard AM Wandell PE Holzmann MJ, et al Barriers and facilitators to participation in a health check for cardiometabolic diseases in primary care: a systematic review. Eur J Prev Cardiol 2018; 25: 1326–1340. 29916723 10.1177/2047487318780751 PMC6097107

[23] Lindholt JS Sogaard R. Population screening and intervention for vascular disease in Danish men (VIVA): a randomised controlled trial. Lancet 2017; 390: 2256–2265. 28859943 10.1016/S0140-6736(17)32250-X

[24] Curry SJ Krist AH Owens DK, et al Screening for atrial fibrillation with electrocardiography: US Preventive Services Task Force recommendation statement. JAMA 2018; 320: 478–484. 30088016 10.1001/jama.2018.10321

[25] King SFA, Bartlett C, Mahon J, et al. Evidence summary for screening for atrial fibrillation in adults. In: *External review against programme appraisal criteria for the UK National Screening Committee*. London: UK National Screening Committee, 2019.

